# INTERNAL MIGRATION AND HEALTH CHANGES: A LONGITUDINAL STUDY OF CHINESE ADULTS IN MID AND LATER LIFE

**DOI:** 10.1093/geroni/igz038.2930

**Published:** 2019-11-08

**Authors:** Li Gao, Zheng Wu, Shu z Li

**Affiliations:** 1 School of Public Policy and Administration, Xi’an, Shaanxi, China; 2 Department of Gerontology, Vancouver, Canada; 3 Institute for Population and Development Studies, Xi’an, Shannxi, China

## Abstract

Objectives: This study examines the effects of internal migration on health status and health changes among middle-aged and older migrants in China. Methods: Using longitudinal data from the 2011-2015 China Health and Retirement Longitudinal Study (CHARLS), this study compares non-migrants with those of recent migrants and earlier migrants in regard to their self-rated health and mental health. OLS and a series of fixed effects models were conducted to examine the effects of migration on health status and health changes. Results: Compared with non-migrants, earlier migrants report better self-rated health but no difference in depression. Our findings demonstrate that recent migrants show better self-rated health changes than non-migrants. In addition, for recent migrants, there are significant changes in self-rated health among rural-to-urban migrants and rural-to-rural migrants, while urban-to-rural migrants and urban-to-urban migrants are not significantly different from non-migrations. Discussion: There are associations between internal migration and self-rated health in China. The effects of migration on health appear to differ by the type of migration. Those who migrated from rural area are mostly likely to be affected by migration. However, migrants from urban area are less affected.

